# Dual deficiency of angiotensin‐converting enzyme‐2 and Mas receptor enhances angiotensin II‐induced hypertension and hypertensive nephropathy

**DOI:** 10.1111/jcmm.15914

**Published:** 2020-09-24

**Authors:** Jun Ni, Fuye Yang, Xiao‐Ru Huang, Jinxiu Meng, Jiaoyi Chen, Michael Bader, Josef M. Penninger, Erik Fung, Xue‐Qing Yu, Hui‐Yao Lan

**Affiliations:** ^1^ Department of Medicine & Therapeutics Li Ka Shing Institute of Health Sciences Lui Che Woo Institute of Innovative Medicine The Chinese University of Hong Kong Hong Kong SAR China; ^2^ Department of Immunology and Microbiology Shanghai Institute of Immunology Shanghai Jiao Tong University School of Medicine Shanghai China; ^3^ Department of Nephrology The Second Affiliated Hospital of Zhejiang University School of Medicine Hangzhou China; ^4^ Guangdong‐Hong Kong Joint Laboratory on Immunological and Genetic Kidney Diseases Guangdong Provincial Key Laboratory Coronary Heart Disease Prevention Guangdong Cardiovascular Institute Guangdong Provincial People’s Hospital Guangdong Academy of Medical Sciences Guangzhou China; ^5^ Max‐Delbrück Center for Molecular Medicine in the Helmholtz Association Berlin Germany; ^6^ Institute of Molecular Biotechnology of the Austrian Academy of Sciences Vienna Austria

**Keywords:** ACE2, Ang II, hypertension, hypertensive nephropathy, Mas, NF‐κB, TGF‐β/Smad3

## Abstract

Angiotensin‐converting enzyme‐2 (ACE2) and Mas receptor are the major components of the ACE2/Ang 1‐7/Mas axis and have been shown to play a protective role in hypertension and hypertensive nephropathy individually. However, the effects of dual deficiency of ACE2 and Mas (ACE2/Mas) on Ang II‐induced hypertensive nephropathy remain unexplored, which was investigated in this study in a mouse model of hypertension induced in either ACE2 knockout (KO) or Mas KO mice and in double ACE2/Mas KO mice by subcutaneously chronic infusion of Ang II. Compared with wild‐type (WT) animals, mice lacking either ACE2 or Mas significantly increased blood pressure over 7‐28 days following a chronic Ang II infusion (*P* < .001), which was further exacerbated in double ACE2/Mas KO mice (*P* < .001). Furthermore, compared to a single ACE2 or Mas KO mice, mice lacking ACE2/Mas developed more severe renal injury including higher levels of serum creatinine and a further reduction in creatinine clearance, and progressive renal inflammation and fibrosis. Mechanistically, worsen hypertensive nephropathy in double ACE2/Mas KO mice was associated with markedly enhanced AT1‐ERK1/2‐Smad3 and NF‐κB signalling, thereby promoting renal fibrosis and renal inflammation in the hypertensive kidney. In conclusion, ACE2 and Mas play an additive protective role in Ang II‐induced hypertension and hypertensive nephropathy. Thus, restoring the ACE2/Ang1‐7/Mas axis may represent a novel therapy for hypertension and hypertensive nephropathy.

## 1 INTRODUCTION

Hypertensive nephropathy is a major complication of hypertension and is the main cause of chronic kidney disease (CKD) characterized by progressive renal fibrosis and inflammation.^1^ Angiotensin II (Ang II), the major functional peptide of the renin‐angiotensin system (RAS), has been long recognized as a key mediator in CKD, particularly in hypertension‐associated nephropathy.^1^ It is well accepted that Ang II can be positively regulated by the classical Ang I‐converting enzyme (ACE)/Ang II/Ang II type 1 receptor (AT1) axis, a critical pathway leading to end‐stage renal disease.^2^, ^3^

Accumulating evidence has shown that the pathogenic actions of the ACE/Ang II/AT1 axis can be countered by the angiotensin‐converting enzyme 2 (ACE2)/Ang 1‐7/Mas receptor (Mas) axis. As a homolog of ACE, the monocarboxypeptidase ACE2 can oppose ACE activity via conversion of Ang II to Ang 1‐7, which binds to the Mas receptor to exert opposite effects on Ang II.^4^, ^5^, ^6^ ACE2 is highly expressed in the normal kidney, mainly located in the proximal tubule,^7^ and plays an essential role in cardiovascular and kidney diseases.^8^ ACE2 deficiency is associated with exaggerated kidney injury in mouse models of diabetes, Ang II‐induced hypertension and obstructive nephropathy,^9^, ^10^, ^11^, ^12^, ^13^ whereas treatment with human recombinant ACE2 (hrACE2) can slow the progression of diabetic and hypertensive kidney injury.^14^, ^15^ Ang 1‐7 is an endogenous ligand for the G protein‐coupled receptor Mas.^16^ When Mas is deleted (*Mas* ^–/–^) in mice with the FVB/N genetic background, they have higher blood pressure than their wild‐type (WT) counterpart (*Mas* ^+/+^).^17^ However, a difference in the mean arterial pressure was not observed in *Mas* ^+/+^ and *Mas* ^–/–^ mice with the C57BL/6 background.^18^ Nevertheless, the genetic deletion of Mas leads to glomerular hyperfiltration and microalbuminuria.^19^ However, the role and underlying mechanisms of the ACE2/Ang1‐7/Mas axis in hypertensive nephropathy remain largely unclear, prompting us to investigate using a chronic Ang II infusion model of hypertension in ACE2 knockout (KO), Mas KO and, importantly, in ACE2/Mas double KO mice.

## 2 MATERIALS AND METHODS

### 2.1 Mouse model of Ang II‐induced hypertension in mice lacking ACE2, Mas or ACE2/Mas genes

ACE2 KO and Mas KO mice (both C57BL/6 background) were generated as described previously.^10^, ^18^ To investigate the role of the ACE2/Ang1‐7/Mas axis in hypertension and hypertensive nephropathy, we created the ACE2/Mas double KO mouse by cross‐breeding the ACE2 KO with the Mas KO mice. Double ACE2/Mas KO mice were used after being backcrossed for 8 generations. Both single‐ and double‐gene KO mice were identified by genotyping with primers as described previously.^10^, ^18^ Hypertensive nephropathy was induced in WT, ACE2 KO, Mas KO and ACE2/Mas double KO mice (male; 8‐10 weeks of age; 20‐25 g) by subcutaneous infusion of Ang II at a dose of 1.0 mg/kg per day or saline as the control for 28 days via osmotic mini‐pumps (Model 2004; ALzet, California).^20^, ^21^ Blood pressure (BP) was measured by the tail‐cuff method using the CODA non‐invasive blood pressure system (Kent Scientific, Torrington, CT) in conscious mice according to the manufacturer's instructions. Each mouse was preconditioned to the blood pressure monitoring procedure for at least 5 cycles before the systolic BP was recorded. The value of systolic BP was the average of 10 readings. Mice were euthanized by exsanguination under anesthesia with pentobarbitone (100 mg/kg i.p.) at day 28 after Ang II infusion. Kidney tissues were collected, and the renal cortex was dissected and used for Western blot and real‐time polymerase chain reaction (PCR) analyses. The experimental procedures were approved by the Animal Experimental Ethics Committee at The Chinese University of Hong Kong.

### 2.2 Proteinuria and renal function analysis

Twenty‐four‐hour urine was collected at day 28 after Ang II infusion for urinary protein measurement using the Quick Start^TM^ Bradford Dye Reagent (Bio‐Rad Laboratories, CA, USA). Serum creatinine (Scr) and urinary creatinine were detected by an Enzymatic Creatinine LiquiColor Reagent kit (Stanbio Laboratory, Boerne, TX, USA) according to the manufacturer's instructions. The creatinine clearance was calculated using the following formula: [urine creatinine (μmol/L) × 24 h urine volume (ml)]/[24 × 60 (min) × serum creatinine (μmol/L)].

### 2.3 Immunohistochemistry

Changes in renal morphology were examined in periodic acid Schiff (PAS)‐stained paraffin sections (4 μm), and glomerular mesangial matrix expansion was scored by counting 30 glomeruli on PAS‐stained section and expressed as 0‐4 point scale (0 = normal; 1 = mild, <25%; 2 = moderate, 25%‐50%; 3 = severe, 50%‐75%; 4 = glomerulosclerosis, >75%). Immunohistochemistry was performed in paraffin sections (3 μm) using the microwave‐based antigen‐retrieval method.^20^, ^21^ The primary antibodies used in the present study were as follows: anti‐collagen I (#1310‐01, Southern Technology, Birmingham, AL, USA), anti‐F4/80 (MCA497, Serotec, Oxford, UK), anti‐phospho‐Smad3 (#600‐401‐919, Rockland Immunochemicals, Gilbertsville, PA, USA), anti‐phospho‐p65 (ab47395, Abcam, Cambridge, MA, USA) and anti‐monocyte chemoattractant protein‐1 (MCP‐1) (sc‐1304, Santa Cruz Biotechnology, Santa Cruz, CA, USA). Positive signals were measured by the Image‐Pro Plus 6.5 quantitative image analysis system (Media Cybernetics, Rockland, MD, USA) as described previously.^20^, ^21^ Briefly, the positive immunostaining signal was selected from the stained kidney tissue sections, and quantitatively measured and expressed as the percentage of the area of the field examined, and at least 8 fields of views were quantified for each section. F4/80‐positive, phospho‐Smad3‐positive and phospho‐p65‐positive cells were enumerated under high‐power fields (×40) by means of a 0.0625‐mm^2^ graticule fitted in the eyepiece of the microscope and expressed as cells/mm^2^, and 10 to 20 fields of views were quantified for each section.

### 2.4 Western blot analysis

Renal cortical tissues were collected for Western blot analysis as described previously.^20^, ^21^ The following antibodies were used: anti‐alpha‐smooth muscle actin (α‐SMA) (M0851, DAKO); anti‐collagen I (#1310‐01, Southern Technology, Birmingham, AL, USA); anti‐phospho‐p44/42 MAPK (ERK1/2) (Thr202/Tyr204) (#4376), anti‐phospho‐Smad3 (Ser423/Ser425) (#9520), anti‐phospho‐IκBα (Ser32) (#2859), anti‐IκBα (#9242), anti‐phospho‐p65 (Ser536) (#3031) and anti‐p65 (#6956) (Cell Signaling Technology, Danvers, MA, USA); anti‐interleukin (IL)‐1β (sc‐7884), anti‐AT1 (sc‐1173), anti‐Smad ubiquitination regulatory factors 2 (Smurf2) (sc‐25511) and anti‐Smad7 (sc‐7004) (Santa Cruz Biotechnology); anti‐Smad3 (#511500, Invitrogen ‐ Thermo Fisher Scientific, Carlsbad, CA, USA); anti‐glyceraldehyde‐3‐phosphate dehydrogenase (GAPDH) (MAB374, Chemicon, Temecula, CA, USA); and LI‐COR IRDye 800‐labelled secondary antibodies (Rockland Immunochemicals). The signals were detected using an Odyssey Infrared Imaging System (Li‐COR Biosciences, Lincoln, NE, USA) and quantified using the ImageJ program (http://imagej.nih.gov/ij/). The ratio of the protein examined was normalized against GAPDH and expressed as a mean ± standard error of the mean (SEM).

### 2.5 Real‐time PCR analysis

Total RNA was extracted from kidney tissues using TRIzol^®^, and mRNA levels of collagen I, α‐SMA, IL‐1β, renin, angiotensinogen, aminopeptidase A (APA) and aminopeptidase N (APN) were detected by real‐time PCR as described previously. The PCR primer sequences were listed in Table 1. The housekeeping genes GAPDH were used as internal controls. The ratio of the mRNA examined to GAPDH was calculated and is expressed as mean ± SEM.

**Table 1:** Real‐time PCR primer sequences

Genes (mouse)	Forward (5’‐3’)	Reverse (5’‐3’)
Renin	CTGTGGGTGGAATCACTGTG	CCAGTGTCCACCACTACCG
angiotensinogen	AGAAGACCCTGCATGATCAAC	TTTTCTCAGTGGCAAGAACT
APA	TTCACATCCAGTGTTCGTCA	TGGAGAGAGCCTCGGCTATCCAATCCCACGTTCC
APN	GCAGAGATGGCACTCCTGGA	CCCTTCAGCTCCTGTCATTCC
Collagen I	GAGCGGAGAGTACTGG ATCG	TACTCGAACGGGAATCCA TC
α‐SMA	GTCCCAGACATCAGGGAGT AA	TCGGATACTTCAGCGTCA GGA
IL‐1β	CTTCAGGCAGGCAGTAT CACTCAT	TCTAATGGGAACGTCACAC ACCAG
GAPDH	GCATGGCCTTCCGTGTTC	GATGTCATCATACTTGGCAG GTTT

Abbreviations: APA, aminopeptidase A; APN, aminopeptidase N.

### 2.6 Statistical analysis

Data obtained from this study were expressed as mean ± SEM. Statistical analyses were performed using one‐way ANOVA using Prism 5.0 (GraphPad Software, San Diego, CA, USA).

## 3 RESULTS

### 3.1 Double deficiency of ACE2/Mas genes significantly increases blood pressure and impairs renal functions in response to Ang II infusion

**Figure 1:**
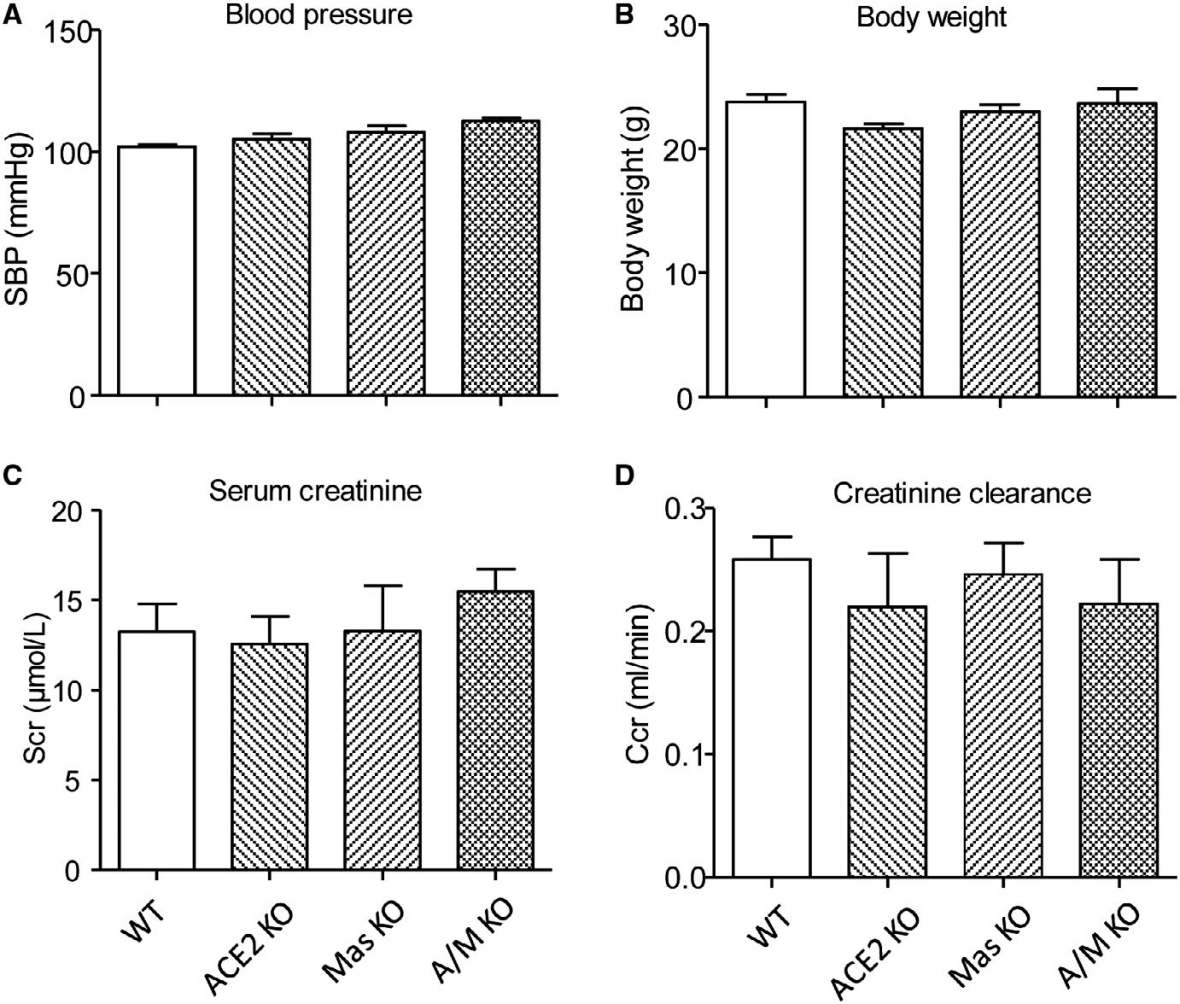
Mice with double deletion of angiotensin (Ang)‐converting enzyme‐2 (ACE2) and Mas genes develop normally with a normal range of blood pressure and renal function. A, Systolic blood pressure (SBP). B, Bodyweight. C, Serum creatinine (Scr). D, Creatinine clearance (Ccr). Data represent the mean ± SEM for groups of 3‐5 mice

**Figure 2:**
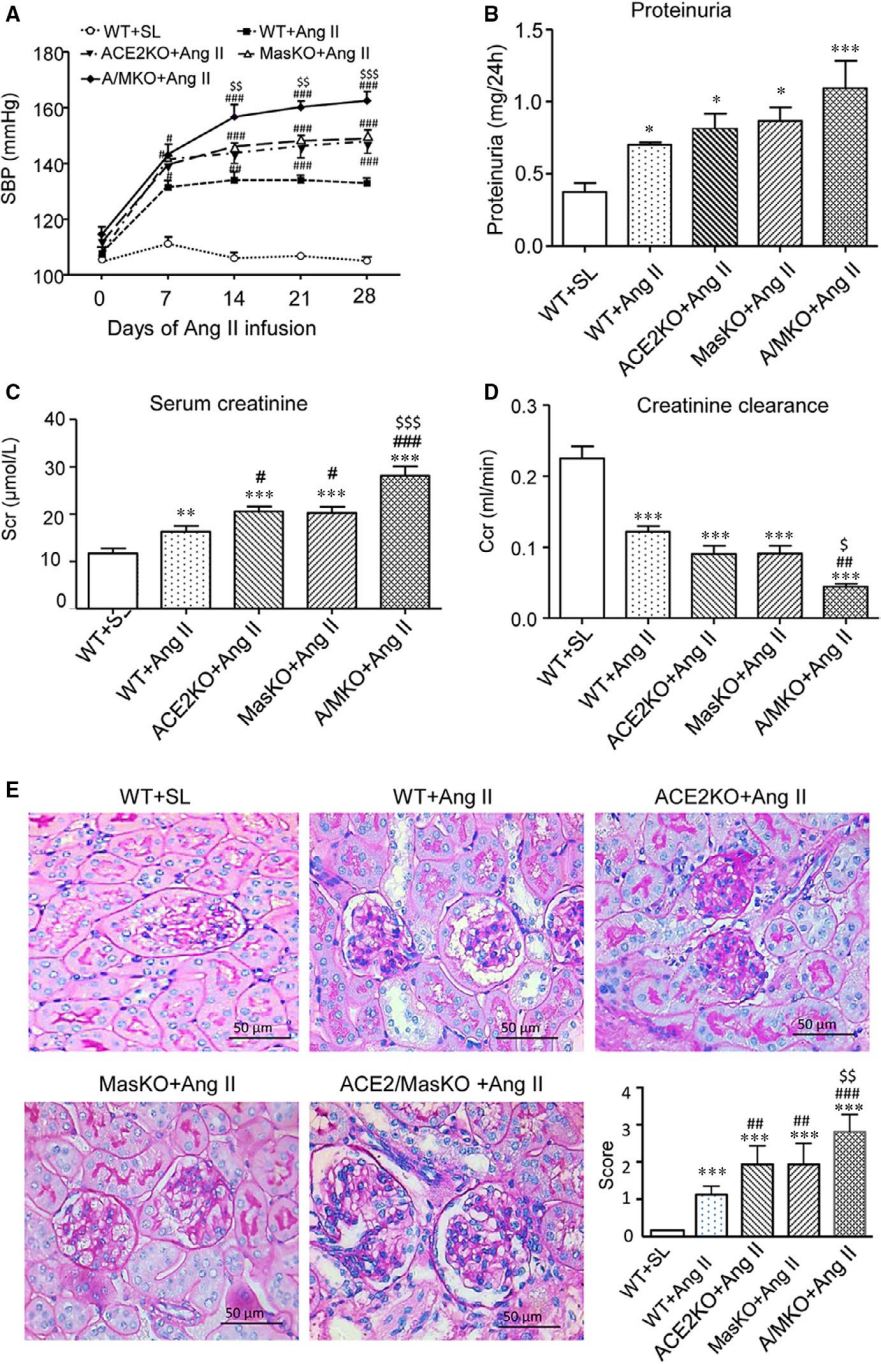
Double deletion of angiotensin (Ang)‐converting enzyme‐2 (ACE2) and Mas receptor enhances Ang II‐induced hypertension and hypertensive renal injury. A, Systolic blood pressure (SBP) at weeks 1, 2, 3, and 4. B, 24‐hour proteinuria. C, Serum creatinine (Scr). D, Creatinine clearance rate (Ccr). E, Renal periodic acid Schiff (PAS) staining at week 4 after Ang II infusion. Glomerular mesangial matrix expansion was scored. Data represent the mean ± SEM for groups of 6‐15 mice. *P < .05, **P < .01, ***P < .001 vs. WT + saline (WT + SL); ^#^ *P* < .05, ^##^ *P* < .01, ^###^ *P* < .001 vs. WT + Ang II; ^$^ *P* < .05^, $$^ *P* < .01, ^$$$^ *P* < .001 vs. ACE2 KO + Ang II and Mas KO + Ang II. Scale bar, 50 μm. WT + SL represents wild‐type (WT) mice received saline infusion; WT + Ang II represents WT mice received Ang II infusion; ACE2 KO + Ang II represents ACE2 KO mice received Ang II infusion; Mas KO + Ang II represents Mas KO mice received Ang II infusion and A/M KO + Ang II represents mice with double deletion of ACE2 and Mas received Ang II infusion

Compared with WT animals, mice with single deletion of ACE2 or Mas, or double deletion of ACE2/Mas developed normally without detectable abnormalities in blood pressure (Figure 1A), bodyweight (Figure 1B), or renal function as determined by serum creatinine (Scr, Figure 1C) and creatinine clearance (Ccr, Figure 1D). All mice receiving chronic Ang II infusion developed hypertension (Figure 2A), elevated Scr (Figure 2C) and reduced Ccr (Figure 2D), accompanied by a significant increase in proteinuria (Figure 2B). In contrast, mice lacking either ACE2 or Mas developed more severe hypertension and renal functional injury, which were further enhanced in mice with double deletion of ACE2/Mas (Figure 2A‐D). Chronic Ang II infusion also caused a moderate glomerular hypercellularity and mesangial matrix expansion in the WT mouse kidneys, which were further increased in mice lacking either ACE2 or Mas gene and became more severe in those with double deletion of ACE2 and Mas (Figure 2E).

### 3.2 Double deletion of ACE2/Mas genes promotes Ang II‐induced renal fibrosis and inflammation

**Figure 3:**
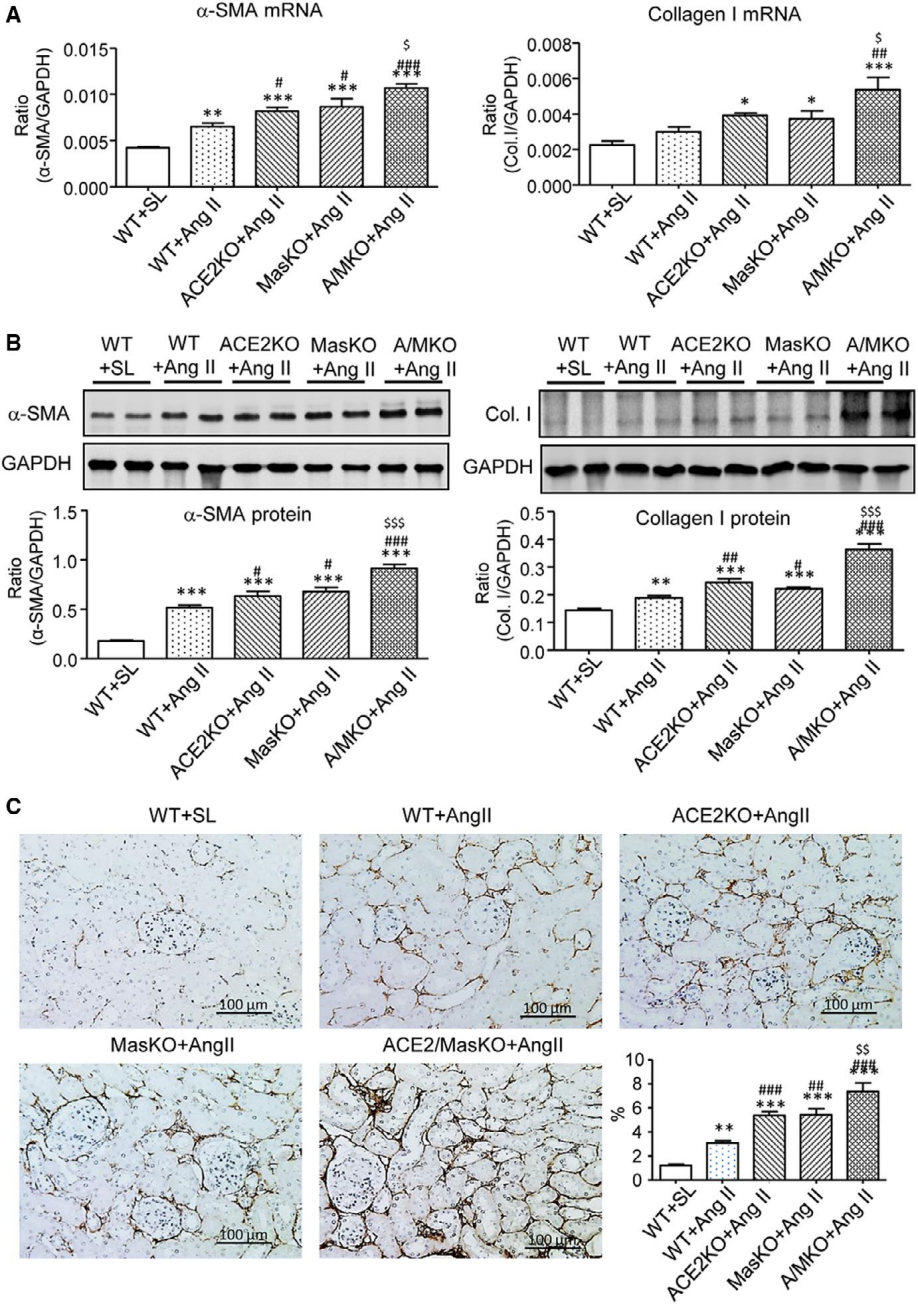
Double deletion of ACE2 and Mas receptor enhances Ang II‐induced renal fibrosis. A, Real‐time PCR detects α‐smooth muscle actin (α‐SMA) and collagen I (Col. I) mRNAs. B, Western blot analysis of α‐SMA and Col. I. C, Col. I deposition detected by immunohistochemistry. Data represent the mean ± SEM for groups of 5‐12 mice. *P < .05, **P < .01, ***P < .001 vs. WT + SL; ^#^ *P* < .05, ^##^ *P* < .01, ^###^ *P* < .001 vs. WT + Ang II; ^$^ *P* < .05, ^$$^ *P* < .01, ^$$$^ *P* < .001 vs. ACE2 KO + Ang II and Mas KO + Ang II. Scale bar, 100 μm

Chronic Ang II infusion resulted in a moderate increase in the expression of α‐SMA and collagen I mRNA and protein in the WT mouse kidneys, as demonstrated by real‐time PCR, Western blot analysis (Figure 3A,B). These fibrotic changes were significantly enhanced in Ang II‐infused mice that lacked either ACE2 or Mas gene. Mice with double deletion of ACE2/Mas developed greater amounts of renal fibrosis indicated by increases in α‐SMA and collagen I mRNA and protein expression when compared with WT and single ACE2 or Mas gene KO mice (Figure 3A,B). Immunohistochemistry also revealed that ACE2/Mas double deficiency further accelerated the accumulation of collagen I in the tubulointerstitium (Figure 3C).

**Figure 4:**
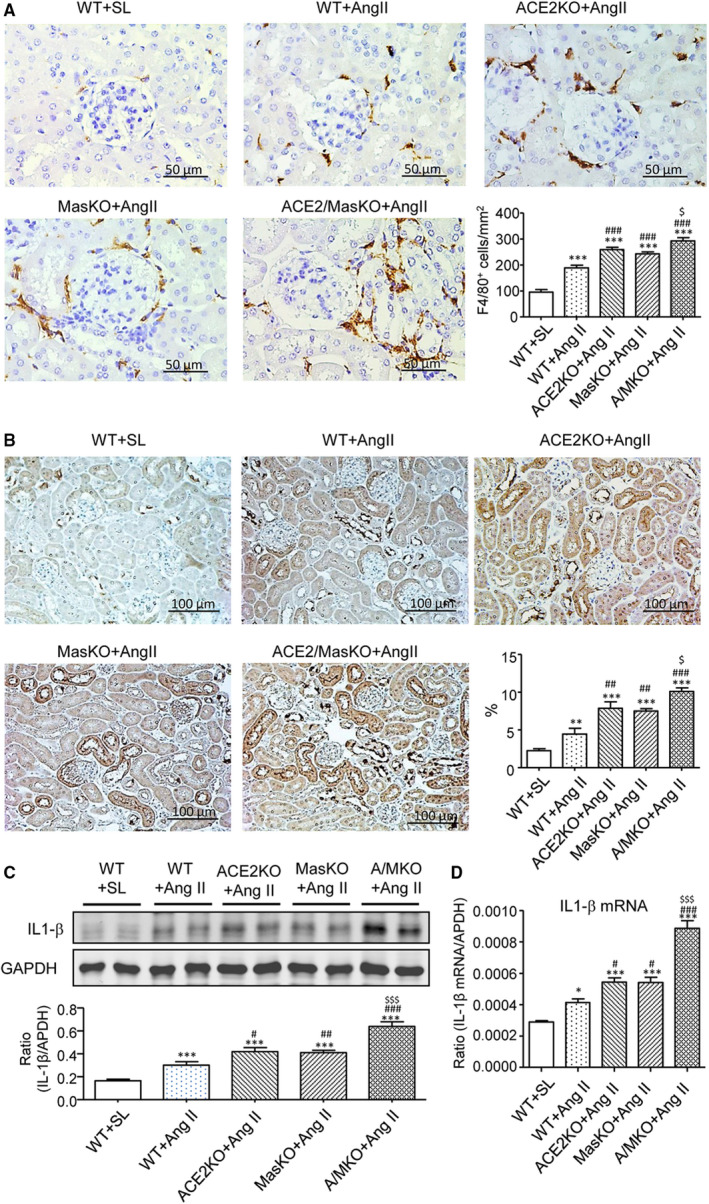
Double deletion of ACE2 and Mas receptor enhances Ang II‐induced renal inflammation. A, F4/80^+^ macrophages infiltrating the kidney detected by immunohistochemistry. B, Monocyte chemoattractant protein‐1 (MCP‐1) expression in the kidney detected by immunohistochemistry. C, Western blot and Real‐time PCR (D) analysis of Interleukin (IL)‐1β expression. Data represent the mean ± SEM for groups of 6‐12 mice. *P < .05, ***P < .001 vs. WT + SL; ^#^ *P* < .05, ^##^ *P* < .01, ^###^ *P* < .001 vs. WT + Ang II; ^$^ *P* < .05, ^$$$^ *P* < .001 vs. ACE2 KO + Ang II and Mas KO + Ang II. Scale bar, 50 μm (A) or 100 μm (B)

Immunohistochemistry, real‐time PCR and Western blot analysis detected that chronic Ang II infusion also induced renal inflammation in the kidneys of WT mice as shown by increasing F4/80^+^ macrophage infiltration (Figure 4A) and up‐regulation of MCP‐1 and IL‐1β (Figure 4B‐D). These changes were potentiated in either ACE2 KO or Mas KO mice and to an even greater extent in mice with double deficiency of the ACE2 and Mas genes (Figure 4).

### 3.3 Double deletion of ACE2/Mas genes promotes Ang II‐induced renal fibrosis by enhancing AT1‐ERK1/2 MAPK‐Smad3 signalling in the hypertensive kidneys

**Figure 5:**
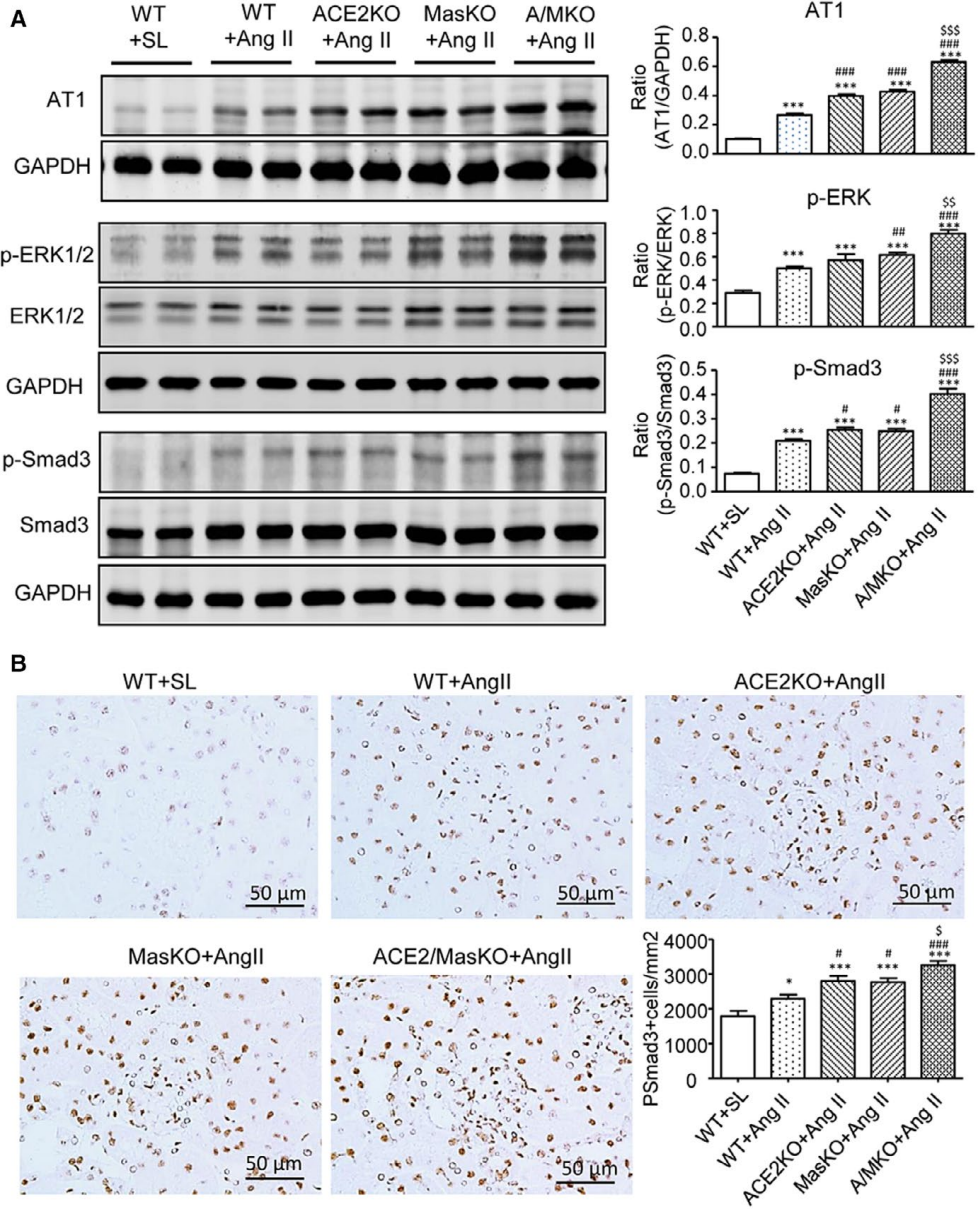
Double deletion of ACE2 and Mas receptor enhances Ang II‐induced activation of AT1, ERK1/2, and Smad3 signalling in the hypertensive kidney. A, Western blot analysis of Ang II receptor type 1 (AT1), phospho‐ERK1/2 (p‐ERK1/2), phospho‐Smad3 (p‐Smad3). B, Immunohistochemistry of p‐Smad3 nuclear translocation. Data represent the mean ± SEM for groups of 6‐12 mice. *P < .05, ***P < .001 vs. WT + SL; ^#^ *P* < .05, ^##^ *P* < .01, ^###^ *P* < .001 vs. WT + Ang II; ^$^ *P* < .05, ^$$^ *P* < .01, ^$$$^ *P* < .001 vs. ACE2 KO + Ang II and/or Mas KO + Ang II. Scale bar, 50 μm

We then examined the possible mechanisms related to renal fibrosis in hypertensive mice with either ACE2 or Mas KO or their double KO. It has been well documented that Smad3 signalling is critical for Ang II‐induced renal and cardiac fibrosis via both TGF‐β‐dependent and AT1‐ERK1/2‐Smad3 crosstalk pathways.^20^, ^21^, ^22^, ^23^, ^24^, ^25^ Thus, we investigated whether deficiency of ACE2 and/or Mas exacerbated Ang II‐induced renal fibrosis via the AT1‐ERK1/2‐Smad3 signalling pathway. Western blot analysis demonstrated that a chronic Ang II infusion increased the expression of AT1 receptors and up‐regulated ERK1/2 and Smad3 signalling as indicated by increased phospho‐ERK1/2 and phospho‐Smad3, respectively, in the kidneys of WT mice (Figure 5A). These changes were significantly increased in mice with single deletion of ACE2 or Mas, and much greater in mice with double deficiency of ACE2/Mas. Immunohistochemistry also detected that either ACE2 or Mas deficiency enhanced Ang II‐induced phospho‐Smad3 nuclear translocation in the hypertensive kidney, which was further increased in mice lacking both ACE2 and Mas genes (Figure 5B).

**Figure 6:**
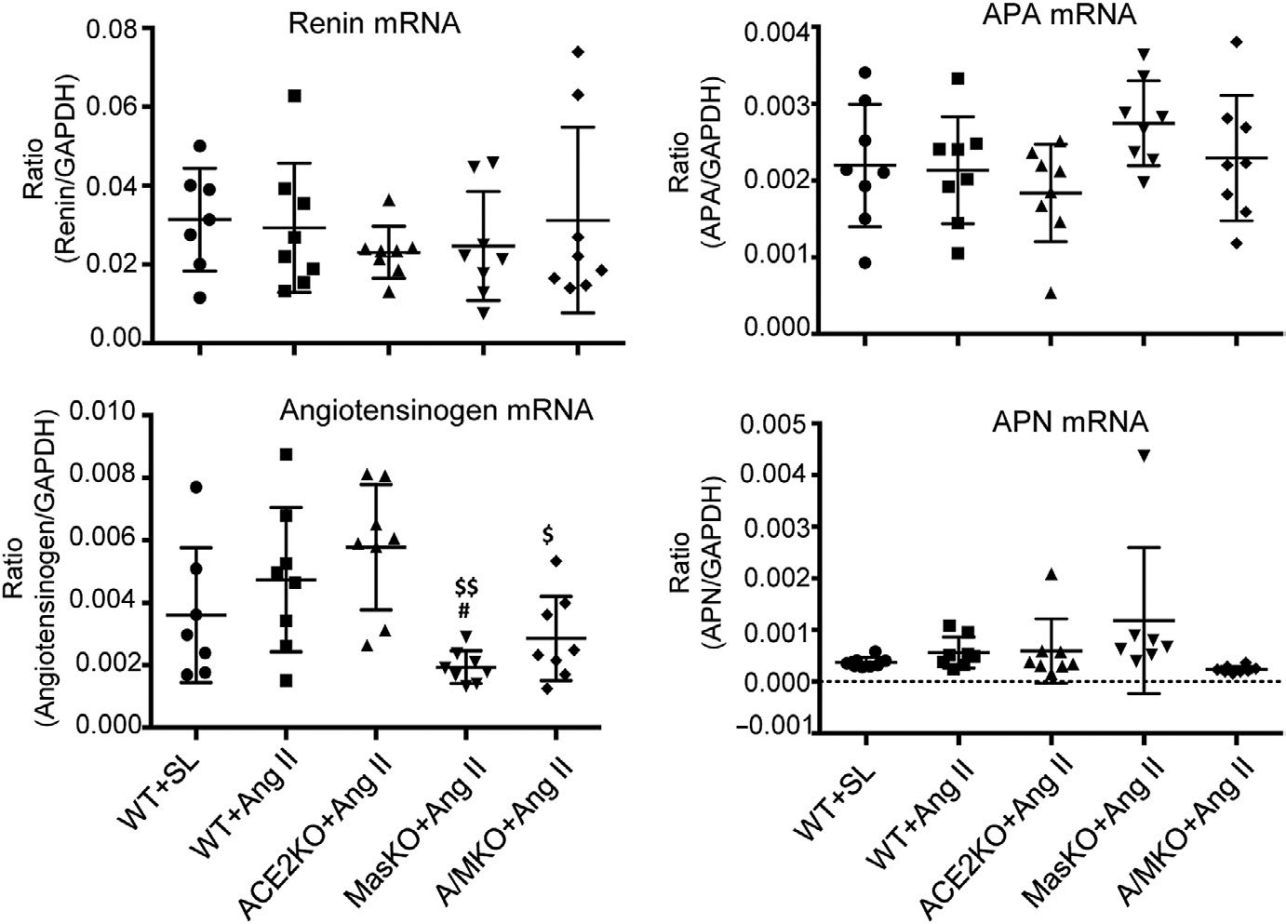
Double deficiency of ACE2 and Mas genes inhibits angiotensinogen but not Renin, Angiotensinogen, Aminopeptidase A mRNA expression in the hypertensive kidney. Renal mRNA expression of Renin, Angiotensinogen, Aminopeptidase A (APA) and Aminopeptidase N (APN) is detected by Real‐time PCR. Data represent the mean ± SEM for groups of 8 mice. ^#^ *P* < .05 vs. WT + Ang II; ^$^ *P* < .05, ^$$^ *P* < .01 vs. ACE2 KO + Ang II and/or Mas KO + Ang II

The other components of RAS, including renin, angiotensinogen, aminopeptidase A (APA) and aminopeptidase N (APN), were also investigated in mice with a chronic infusion of Ang II. Real‐time PCR revealed that a single or double deletion of ACE2 and Mas had no effect on renin, APA and APN mRNA expression, whereas angiotensinogen mRNA expression was significantly reduced in Mas or double ACE2/Mas KO mice but remained unchanged in ACE2 KO mice (Figure 6).

### 3.4 Double deletion of ACE2/Mas genes promotes Ang II‐induced renal inflammation via Smurf2‐mediated loss of smad7 dependent activation of NF‐κB signalling

**Figure 7:**
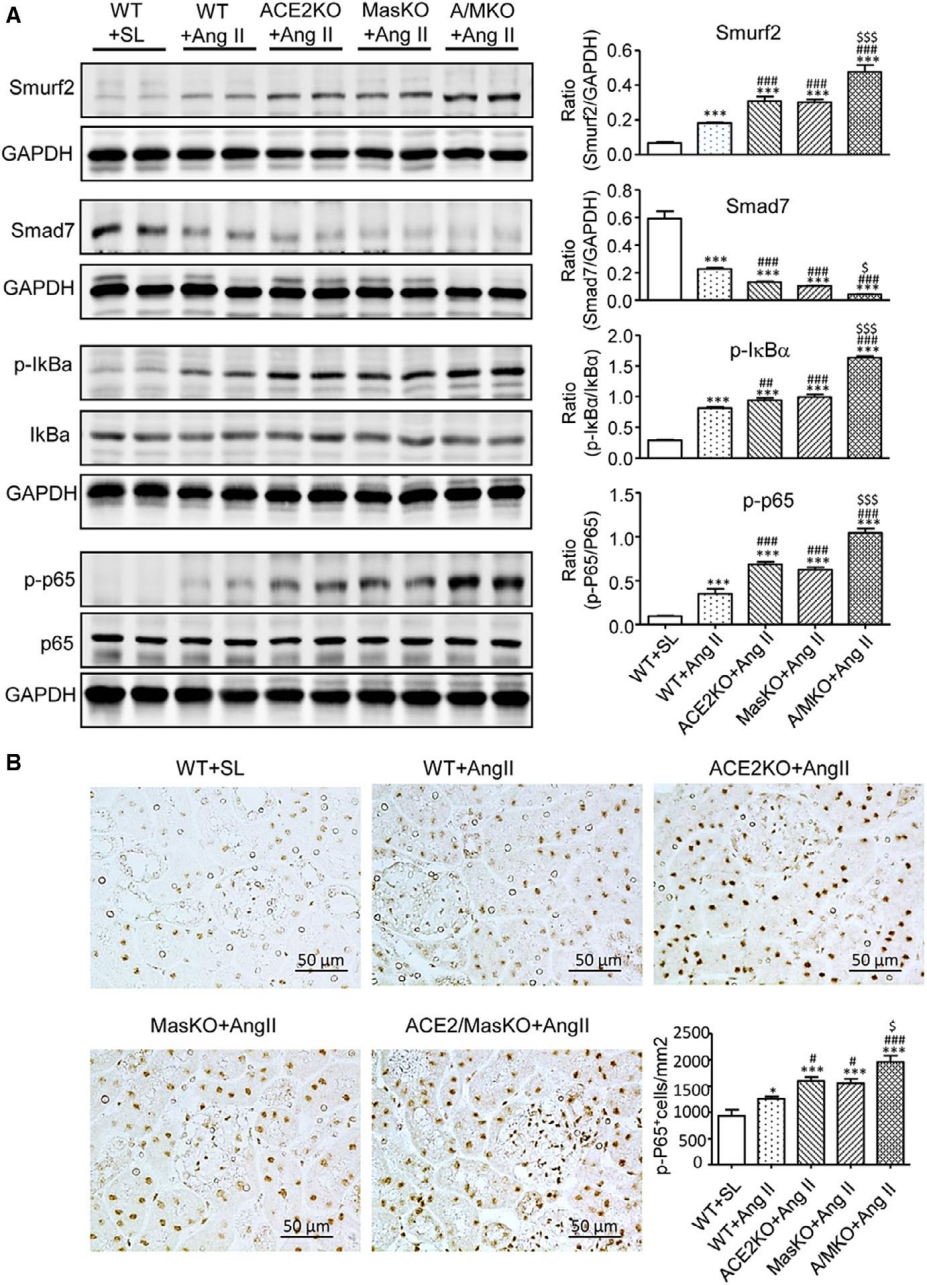
Double deletion of ACE2 and Mas receptor enhances Ang II‐induced activation of nuclear factor κB (NF‐κB) signalling in the hypertensive kidney. A, Western blot analysis of Smad ubiquitination regulatory factors 2 (Smurf2), Smad7, phospho‐IκBα (p‐IκBα), and phospho‐NF‐κB/p65 (p‐p65). B, Immunohistochemistry of NF‐κB/p‐p65 nuclear translocation. Data represent the mean ± SEM for groups of 6‐12 mice. *P < .05, ***P < .001 vs. WT + SL; ^#^ *P* < .05, ^##^ *P* < .01, ^###^ *P* < .001 vs. WT + Ang II; ^$^ *P* < .05, ^$$$^ *P* < .001 vs. ACE2 KO + Ang II and Mas KO + Ang II. Scale bar, 50 μm

We have previously demonstrated that Smad7, an inhibitor of TGF‐β/Smad signalling, can also inactivate Ang II‐induced NF‐κB signalling through inducing IκBα, an NF‐κB inhibitor.^26^, ^27^, ^28^, ^29^ We also find that Ang II can induce an E3‐ligase, Smurf2, which can bind and degrade Smad7, thereby promoting Smad3‐dependent renal fibrosis and NF‐κB‐mediated renal inflammation.^20^, ^24^ Similarly, the present study detected that deletion of ACE2 or Mas enhanced Ang II‐mediated up‐regulation of renal Smurf2, which was associated with a loss of renal Smad7, resulting in activation of NF‐κB signalling by increasing phospho‐IκBα and phospho‐NF‐κB/p65 subunit levels and phospho‐NF‐κB/p65 nuclear translocation (Figure 7). These changes were notably enhanced in the diseased kidney of dual ACE2/Mas KO mice (Figure 7). Thus, double deletion of ACE2/Mas enhanced Ang II‐induced renal inflammation via the Smurf2‐mediated loss of Smad7‐dependent NF‐κB signalling.

## 4 DISCUSSION

In this study, we found that mice with a single or double deletion of ACE2 and Mas gene developed normally with a normal range of blood pressure, bodyweight and renal function. However, under hypertensive conditions, a single deletion of ACE2 or Mas gene significantly enhanced Ang II‐induced hypertension and hypertensive nephropathy, which was consistent with the known protective roles of ACE2 or Mas in attenuating the detrimental effects of hypertension and hypertensive kidney disease.^9^, ^13^, ^30^, ^31^, ^32^ Indeed, ACE2/Ang 1‐7/Mas is considered as a protective pathway to counter‐regulate the pathogenic action of ACE/Ang II/AT1 axis. The binding of Ang 1‐7 to Mas receptor can antagonize the effects of Ang II.^4^ Furthermore, Mas can hetero‐oligomerize with the AT1 to form a constitutive complex which is stable in the presence of antagonists or agonists of AT1 and Mas. Co‐expression of Mas in CHO cells can reduce AT1‐mediated intracellular calcium mobilization and inositol phosphate generation, indicating that Mas itself can inhibit the actions of Ang II..^33^

A most significant finding from this study was that compared to either ACE2 or Mas KO mice, mice with double deletion of ACE2 and Mas genes developed more severe hypertension and hypertensive nephropathy including higher levels of blood pressure and serum creatinine, a significant fall of creatinine clearance, and progressive renal inflammation and fibrosis. These observations suggested that ACE2 and Mas may work additively to protect against Ang II‐induced hypertension and hypertensive kidney disease.

Enhanced AT1‐ERK1/2‐Smad3 signalling could be a key mechanism by which the dual deletion of ACE2 and Mas further promoted renal fibrosis in the hypertensive kidney in response to Ang II. It is now well accepted that Smad3 is a key profibrotic transcription factor and plays an essential role in cardiovascular and renal fibrosis under high Ang II conditions.^20^, ^21^, ^22^, ^23^, ^24^, ^25^ Indeed, Ang II is able to directly and indirectly activate Smad3 to cause cardiovascular and renal fibrosis via a TGF‐β‐dependent and ERK1/2‐Smad crosstalk pathway.^20^, ^21^, ^22^, ^23^, ^24^, ^25^ We have previously reported that Ang II can induce a rapid activation of Smad2/3 at 15 minutes, which results in a subsequent expression of collagen I in tubular epithelial cells lacking the *TGF‐β* gene. These profibrotic actions are blocked by the AT1 antagonist (losartan) and ERK1/2 inhibitor (PD98059).^23^ In addition, disruption of Smad3 prevents Ang II‐induced kidney injury by preserving renal function, inhibiting renal fibrosis and inflammation, but has no effect on Ang II‐induced high blood pressure in vivo.^20^ In renal mesangial cells, Ang1‐7 treatment can repress Ang II‐induced ERK1/2 phosphorylation in a dose‐dependent manner.^34^ These findings reveal that Ang II may act via the AT1‐ERK1/2‐Smad3 pathway to mediate renal fibrosis, which can be counter regulated by Ang1‐7. In addition, Ang II can also activate Smad3 by degrading Smad7, a downstream inhibitor of TGF‐β/Smad signalling, via the Smurf2‐dependent ubiquitin‐proteasome degradation mechanism.^20^, ^24^, ^27^, ^28^ Thus, deletion of Smad7 promotes but overexpression of Smad7 inhibits Ang II‐induced AT1‐ERK1/2‐Smad3‐mediated hypertensive nephropathy.^27^, ^28^, ^29^ Consistent with these findings, this study demonstrated again that a chronic Ang II infusion led to a moderate activation of AT1‐ERK1/2‐Smad3 signalling and produced a moderate renal fibrosis by increasing accumulation of α‐SMA and collagen I matrix. A single deletion of ACE2 or Mas increased further AT1‐ERK1/2‐Smad3 signalling and renal fibrosis, which became more severe in mice lacking both ACE2/Mas genes. Thus, enhanced AT1‐ERK1/2‐Smad3 signalling may be a mechanism by which loss of the ACE2/Ang 1‐7/Mas axis exacerbated renal fibrosis in ACE2/Mas double KO mice.

It is well established that Ang II acts via AT1 to activate NF‐κB signalling that mediates renal inflammation. Consistent with this notion, the present study found that a single deletion of ACE2 or Mas sustained AT1‐NF‐κB‐driven renal inflammation, which was further increased in mice with ACE2/Mas double deletion. Furthermore, Ang II can also activate NF‐κB by degrading Smad7 as Smad7 is capable of inducing IκBα, an inhibitor of NF‐κBα.^20^, ^21^, ^25^, ^26^, ^27^, ^28^, ^29^ Consistent with these findings, enhanced Ang II‐induced NF‐κB‐dependent renal inflammation in ACE2 KO, Mas KO and ACE2/Mas double KO mice was also associated with increasing Smurf2‐dependent ubiquitin degradation of Smad7.

It should be pointed out that there are several notable limitations in this study. First, the present study was performed in global ACE2 or Mas gene KO mice, and thus, it is difficult to distinguish the systemic versus the local effect of ACE2 and Mas on Ang II‐induced hypertension and hypertensive kidney injury. Second, it has been reported that antibodies against AT1 receptor are non‐specific,^35^, ^36^, ^37^ which may inference the outcome and interpretation of the AT1 results. However, as it has been well established that Ang II acts via AT1 to activate NF‐κB‐driven inflammation and Smad3‐dependent fibrosis, the marked differences in AT1‐associated TGF‐β/Smad3‐mediated renal fibrosis and NF‐κB‐dependent renal inflammation in response to Ang II as seen in the present study remained meaningful. Third, although chronic Ang II infusion did cause a compensatory increase in urinary Ang 1‐7 production to counteract the effect of Ang II in WT mice, which was reduced in mice lacking ACE2 or ACE2/Mas, but not in Mas KO mice, we failed to obtain meaningful levels of Ang 1‐7 in serum and intrarenal tissues due to its low concentrations. Thus, changes in urinary levels of Ang 1‐7 remain unexplained and may require further investigation.

In summary, ACE2 and Mas may function additively to protect against Ang II‐mediated hypertension and hypertensive nephropathy as mice with double deletion of ACE2 and Mas developed more severe hypertension and hypertensive nephropathy when compared to either ACE2 or Mas KO mice. Enhanced AT1‐ERK1/2‐Smad3 signalling may be a key mechanism by which the dual deletion of ACE2 and Mas further promoted hypertension and hypertensive kidney disease. Results from this study reveal that the ACE2/Ang 1‐7/Mas axis represents opportunities for the future development of therapeutics against hypertensive disorders.

## CONFLICT OF INTEREST

The authors declare that there are no competing interests associated with the manuscript.

## AUTHOR CONTRIBUTIONS

JN, FY and XRH performed the experiments, analysed the data and drafted the manuscript. XRH, JM and JC generated ACE2 KO, Mas KO and double ACE2/Mas KO mice and animal model. MB provided Mas KO mice and JMP provided the ACE2 KO mice. EF and XQY edited and revised the manuscript. HYL designed and supervised experiments and revised the manuscript.
