# Correction to Dual deficiency of angiotensin‐converting enzyme‐2 and Mas receptor enhances angiotensin II‐induced hypertension and hypertensive nephropathy

**DOI:** 10.1111/jcmm.17909

**Published:** 2023-08-30

In Jun Ni et al.,^1^ the grant number of Shanghai Municipal Health Commission was incorrect. It should have been corrected from 202040117 to 202040119. The correct funding information and acknowledgements are shown below.
